# Incidence of antibiotic resistance genotypes of *Vibrio* species recovered from selected freshwaters in Southwest Nigeria

**DOI:** 10.1038/s41598-022-23479-0

**Published:** 2022-11-07

**Authors:** Ibukun M. Adesiyan, Mary A. Bisi-Johnson, Anthony I. Okoh

**Affiliations:** 1 Institute of Ecology and Environmental Studies, Obafemi Awolowo University, Ile Ife, Nigeria; 2 South Africa Medical Research Council, Water Monitoring Centre, University of Fort Hare, Alice, South Africa; 3 Department of Environmental and Occupational Health, University of Medical Sciences, Ondo, Ondo State Nigeria; 4 Department of Microbiology, Obafemi Awolowo University, Ile Ife, Nigeria; 5 Department of Environmental Health Sciences College of Health Sciences, University of Sharjah, Sharjah, United Arab Emirates

**Keywords:** Antimicrobials, Environmental microbiology, Microbiology, Environmental sciences

## Abstract

*Vibrio* species are classified as potent hazards because of their tendency to effect serious diseases like cholera and other gastrointestinal ailments in humans, as well as vibriosis in fish. A total of 144 freshwater samples were aseptically collected monthly across four rivers (Asejire, Ona, Dandaru and Erinle rivers) over a 12-month period from which *Vibrio* spp. were isolated using culture procedures, confirmed by means of biochemical test as well as Polymerase Chain Reaction (PCR) assay and further characterized for their phenotypic antibiotic susceptibilities and relevant antimicrobial resistant determinants by PCR. Three hundred and fifteen (58%) isolates confirmed across the sampled sites (Asejire = 75, Dandaru = 87, Eleyele = 72, Erinle = 81) showed high resistance against erythromycin—95%, Sulphamethoxazole—94%, rifampicin—92%, doxycycline—82%, tetracycline—75%, amoxicillin—45%, cephalothin—43% and varied susceptibilities to other antibiotics. The multiple antibiotic resistance indices of 97% of the *Vibrio* isolates were above the 0.2 threshold limit with MAR phenotype pattern E-SUL-RF-TET-DOX (0.38) found to be the most prevalent pattern among the isolates. The distributions of resistance determinant of the tested antibiotics were revealed as follows: *sul*II 33%, *sul*I 19% (sulfonamides); *bla*_*OXA*_ 27%, *amp*C 39%, *bla*_*pse*_ 11% (beta-lactams); *tet*A 28%, *tet*E 20%, *tet*39 8%, (tetracyclines) and *strA* 39%. *aacC*2 24%, *aphA*1 14% (aminoglycosides). Strong positive associations were observed among *tetA, sulI, tetE* and *sul*II. This study raises concerns as these selected rivers may contribute to the environmental spread of waterborne diseases and antibiotic resistance genes. Therefore, we recommend environmental context-tailored strategies for monitoring and surveillance of resistance genes so as to safeguard the environment from becoming reservoirs of virulent and infectious *Vibrio* species.

## Introduction

*Vibrio* species are aquatic bacteria of ecological significance belonging to Vibrionaceae family. It is phenotypically characterized as Gram-positive, oxidase positive, motile rods, halophilic and facultatively anaerobic in its metabolism. They are commonly regarded as autochthonous to marine environment and its detection in freshwater ecosystem has been documented globally. There are over 140 different species of *Vibrios* documented currently^1^, however, 12 *Vibrio* species are classified as infectious to humans with *V. cholerae* recognized as the causative agent of cholera disease in humans. Other non-cholerae *Vibrio* spp. are common causative agents of foodborne related infection in humans arising from contaminated water, undercooked or raw seafood consumption^2^. The common symptom of *Vibrio* infection is gastroenteritis and diarrhea which may sometimes be associated with virulence factors like shiga-like cytotoxin, toxin-coregulated pilus, cytotoxins, siderophores, hemolysins, proteases and heat-stable enterotoxin^3,4^.

The incidence of *Vibrio* associated diseases in human and animal is increasingly being reported globally. This bacterium has developed to inhabit diverse settings within ecosystems due to their complex mode of living^5^. They have been isolated from diverse environments including crustacean guts, fishes in sea hydrothermal vent, rivers, wastewater, plants etc. and they carry out important role of nutrient cycling in aquatic milieus^6–8^.

*Vibrio* infections are commonly treated with tetracyclines, third-generation cephalosporin, fluoroquinolone, trimethoprim-sulfamethoxazole as well as aminoglycoside. Tetracyclines and fluoroquinolones are mostly used in protracted or severe cases of *Vibrio* illnesses as they are known to be associated with lesser mortality rates^4^. However, in recent decades, many bacteria including *Vibrio* have emerged with unprecedented resistance to many antimicrobials owing to abusive use in agricultural and human systems^9^.

Antibiotics is among a prominent member of the list of “contaminants of emerging concerns” circulating in different environments. Bacteria that are exposed to antibiotics in aquatic environments can become antibiotic resistance^10^ and their resistance genes can be transferred to other pristine microorganisms by means of mobile genetic elements and horizontal gene transfer thereby **c**ausing changes in the development and adaptation of microbial residents of aquatic environment^11^.

Evidence has shown that anthropogenic activities resulting in agricultural, municipal and aquaculture wastes as well as water run-off can introduce multiple antibiotic resistance organism and their resistance factors into the environment and water bodies^12,13^. Likewise unregulated and ease of access to antibiotics in developing nations like Nigeria can contribute to persistent exposure of humans to antibiotics via consumption of water and food. Thus, polluted aquatic environments have now been identified as significant reservoirs of antibiotic resistance genes requiring more investigation^14^. Some resistance genes contribute to the virulence expression in *Vibrio* infections in human and animal setting and this includes beta lactam genes (*blaOXA, ampC*), sulfonamides genes (*sul1 and sul2*), quinoline genes (*tetA, tetE*) etc.

In most developing countries, identification of new and emerging threats, practices of good treatment of patients, and effective control of antibiotic resistance is not effective due to non-existence of national antimicrobial resistance surveillance programs. Even where surveillance programs do exist, implementation strategies are lacking due to poor infrastructural and equipment support^15^. Hence, monitoring antibiotic resistance patterns of pathogenic bacteria in anthropogenically impacted environment is a relatively cost effective substitute where a national surveillance activity is absent^16^.

Moreover, if the dynamics of antibiotic resistance in pathogenic organisms and diversity of their resistance genes are to be accurately evaluated; there is a need to assess the freshwater environment for contamination with pathogens such as *Vibrio* spp. and clinically relevant antibiotic resistance genes. Therefore, we document the first assessment of the prevalence of 11 resistance determinants of *Vibrio* isolates obtained from four selected freshwater bodies in South-western region of Nigeria.

## Methodology

### The study area

**Figure 1:**
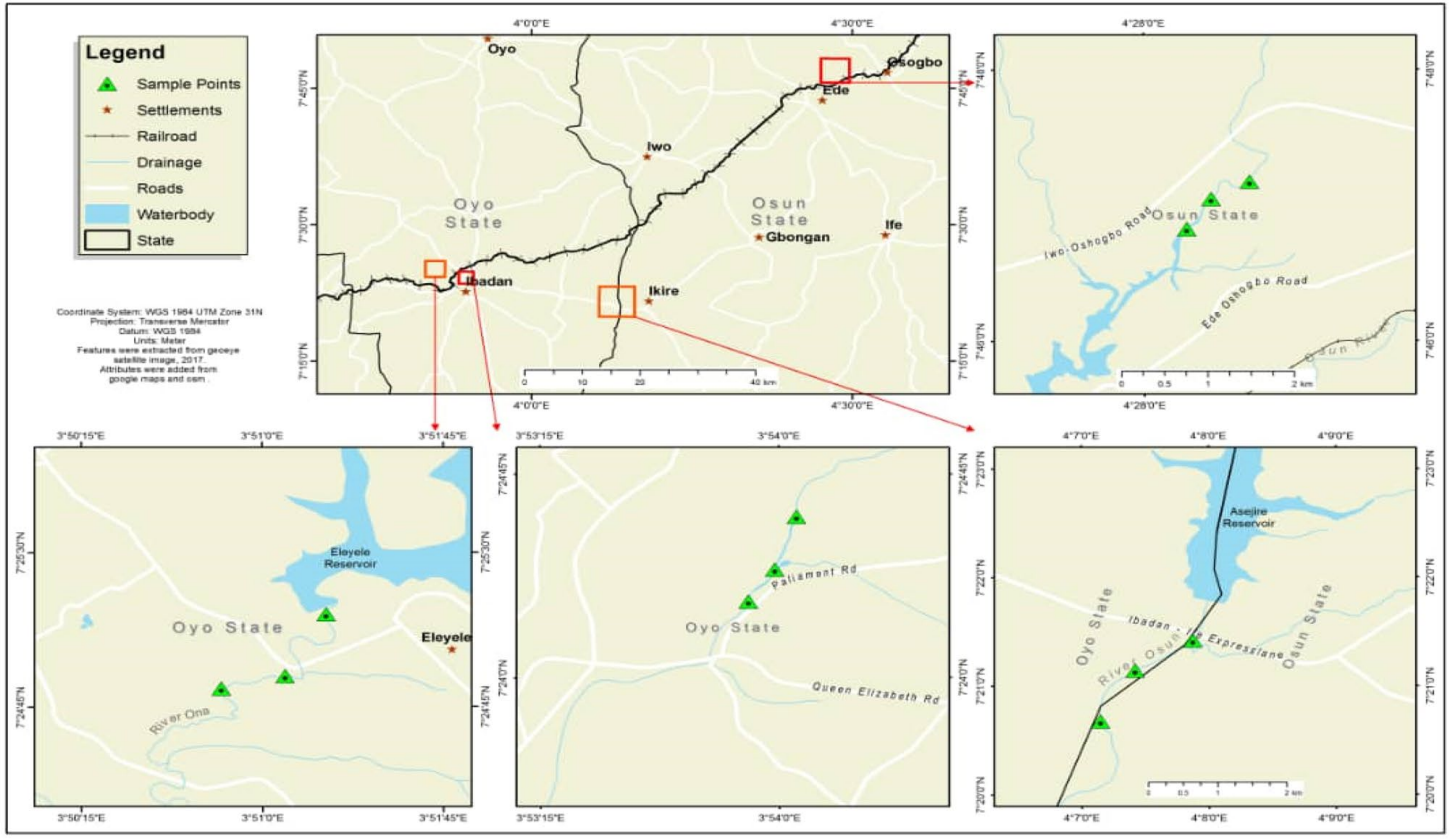
Map showing the four sampled sites.

The sampled rivers selected for this study includes: Asejire—SS1 (N7° 21′ 46.9″ E4° 07′ 51.5″), Dandaru—SS2 (N7° 26′ 25.0″ E3° 49′ 57.5″), Eleyele—SS3 (N7° 26′ 17.1″ E3° 52′ 42.2″), Erinle—SS4 (N7° 46′ 23.7″ E4° 27′ 58.3″); located in three towns in Southwest Nigeria (Ibadan, Asejire and Ede) (Fig. 1). The climate is tropical with rain forest vegetation and atmospheric temperature average of 32 °C. These rivers function as key water source particularly to the rural neighborhoods that borders the cities. The river Ona at Eleyele and River Dandaru at Agodi were selected in Ibadan, for Oyo State. Asejire river, the river at the boundary of Osun and Oyo states (abstracted by Oyo State) and Erinle river in Ede were selected for Osun State. These rivers which serve as collector of effluents from animal, human, hospitals, industries, markets, are often contaminated with organic and inorganic substances from anthropogenic activities with higher concentration during dry seasons. There are a couple of activities around these rivers which includes: markets, small scale farming, animal rearing, active fishing and people were also seen washing clothes and bathing during visit.

### Sampling, enumeration and isolation of presumptive *Vibrio*

Pre-sterilized 1 L plastic bottles were used for water sample collection. After the bottles were triple rinsed with sample water, actual samplings were done at approximately 20 cm below the water surface. Samples were subsequently transported in ice coolers and processed according to the recommendation of the American Public Health Association^16^. By means of standard membrane filtration method, 100 ml each of sampled was filtered through 90 mm, 0.45 μm pore-size filter papers (Millipore, Ireland) and were thereafter transferred into agar plates of TCBS (Thiosulphate Citrate Bile Salts, Conda, Pronasida) and then incubated overnight at 37 °C. Yellow and green characteristic colonies were quantified as colony forming units (CFU) per 100 mL of samples and 3–5 colonies each were randomly selected, Gram-stained and subjected to oxidase test. Afterwards, 315 presumptive *Vibrio* species that were Gram-negative and oxidase positive were purified on a non-selective agar and stored for molecular analysis.

### Extraction of genomic DNA

The isolates' genomic DNA extraction was carried out using the boiling technique^6^. Specifically, 18–24 h old pure colonies of presumptive *Vibrio* spp. were suspended in sterile 200 μl distilled water, then lysed by heating for 15 min at 100 °C temperature using an AccuBlock (Labnet). A Mini Spin microcentrifuge was used to decant cell debris by centrifugation for 10 min at rate of 15,000 rpm. The DNA template from the recovered supernatants was employed in subsequent PCR tests.

### Molecular confirmation of *Vibrio* genus

Genotypic confirmation of *Vibrio* was established by PCR using the *Vibrio* genus primers (F: CGG GAAATGCGTAGAGAT R: TACTAGCGATTCCGAGTTC)^17^ to amplify a 663 bp sequence of the 16s RNA genes of *Vibrio*.

The reaction mixture contains PCR Master Mix (12.5 μl), (Thermo Scientific), oligonucleotide primer (1 μl each) (Inqaba Biotech, SA), DNA template (5 μl), and, nuclease free water (5.5 μl), to make up 25 μl. reaction volume. The PCR protocols used comprises: Initial denaturation for 15 min at 93 °C followed by 35 cycles of denaturation—annealing—elongation steps at 92 °C for 40 s; 57 °C for 1 min and 72 °C for 1.5 min respectively followed by 7 min final extension at 72 °C. Electrophoresis was performed by loading 5 μl amplicons, 3 ul 100-bp ladder (Thermo Scientific), on 2% (w/v) agarose gel (Merck) pre-stained with 5 μl ethidium bromide (Sigma-Aldrich). The electrophoresis process was carried out at 90 V for 50 min and visualized under an ultra-violet trans-illuminator system (Alliance 4.7).

### Antibiotic susceptibility profiling of *Vibrio* isolates

Standards disc diffusion technique with Mueller–Hinton agar (MHA) was used for antimicrobial susceptibility testing^18^, Fresh cultures (18–22 h old) of all isolates that have been positively identified were introduced into 5 mL of 0.85% sterile saline with the suspension turbidity set to 0.5 McFarland standards. MH agar plates were inoculated uniformly with sterile swabs after which discs were aseptically added and incubated for 18–24 h at 37 °C. Measured Zones of inhibition were interpreted as resistant (R), susceptible (S), intermediate (I), using the zone diameter interpretation according to CLSI guideline^18^.

Eighteen (18) antibiotics which comprise the Centre for disease Control (CDC) recommended antibiotics for *Vibrio* infection treatment selected for this assay are: Amikacin—AMK (30 μg), Streptomycin—S (300 μg), Gentamycin—G (10 μg), Cefotaxime—CEF (30 μg), Imipenem—IMI (10 μg), Ciprofloxacin—CIP (5 μg), Meropenem—MEM (10 μg), Ampicillin—AP (10 μg), Sulphamethoxazole—SUL (25 μg), Chloramphenicol—C (30 μg), Tetracycline—TET (30 μg), Erythromycin—E (15 μg), Trimethoprim + Sulphamethoxazole—TS (25 μg), Amoxycillin—AMC (25 μg), Norfloxacin—NOR (30 μg), Doxycycline—DOX (30 μg), Cephalothin—CEP (30 μg) and Rifampin—RF (5 µg).

### Antibiotics resistance phenotyping and indexing

The antibiotic resistance profile of the confirmed *Vibrio* spp. was obtained and those that presented resistance to three and more antibiotics were “phenotyped” to indicate the multiple antibiotic resistances (MAR) phenotypes expressed as:

$$ {\text{MAR}}_{{{\text{index}}}} = {\text{ a/b}} $$

a = total antibiotics that showed resistant, b = total antibiotics selected for the assay^19,20^.

MAR index value ≥ 0.2 threshold suggests high-risk pollution, possibly by uncontrolled use of antimicrobials around the sample sites, which may lead to development of antibiotic resistance^21^. Pattern of antibiotic resistance (ARPA) was likewise evaluated^22^.

$$ {\text{ARPA }} = {\text{ total resistance types/total strains assayed}} $$

### Resistance quotients (RQs) determination

Possible variations in the phenotypes of antimicrobial-resistant *Vibrio* isolates in the selected freshwaters were determined by calculating their resistance quotients^23^. This determines probable ecological-risk presented by antibiotics tested on the confirmed *Vibrio* isolates. The RQs were determined according to the equation^24^:

$$\mathrm{Resistant \; quotient }=\frac{\mathrm{Number \; of \; resistant \; bacteria }}{\mathrm{Total \; number \; of \; bacteria  \; tested}}\times100$$

### PCR detection antibiotic resistance genes of *Vibrio* isolates

Phenotypically resistance *Vibrio* isolates were evaluated for their antibiotic resistant genes using specific primer pairs and PCR conditions (Supplementary Table 1). Resistance genes assayed includes; sulfonamide genes (*sulI, sulII*), tetracycline genes (*tetA, tet39*, *tetE*), β-lactamases genes (*bla*_*OXA,*_* ampC, bla*_*P,SE*_), and aminoglycoside genes (*aacC2, aphA2, strA*). The PCR assay and electrophoresis procedure is as described in “Extraction of genomic DNA” section. Likewise multiple antibiotic resistance genes patterns of all *Vibrio* isolates harboring ≥ 2 resistance genes were determined.

### Statistical analysis

The data obtained were statistically analyzed using IBM (SPSS) version 22.

The associations between the resistance genes found in *Vibrio* isolates were evaluated with Pearson’s chi-square exact test at P = 0.05. A positive relationship between two genes indicates that the genes were associated together, while a negative correlation shows there is no association between the genes.

## Results

### *Vibrio* genus confirmation

**Figure 2:**
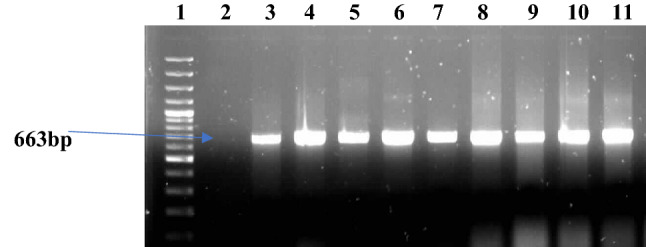
PCR product of the amplification of *toxR* gene for *Vibrio* genus. Confirmation. Lane 1*:* Molecular weight marker (100 bp); lane 2: negative control; lane 3: positive control; lane 4–11*: Vibrio* positive isolates (663 bp).

The counts of *Vibrio* confirmed isolates across the samples freshwaters ranges between 72 CFU/100 ml at SS3 and 87 CFU/100ML at SS2 (Table 1). In total, 58% (315) isolates were positively confirmed by PCR as *Vibrio* (Fig. 2) out of the 548 presumptive isolates confirmed by Gram staining and oxidase test.

**Table 1:** Number of confirmed *Vibrio* isolates.

Site location	Code	*Vibrio* average count (CFU/100 ml)	Confirmed *Vibrio* isolates
Asejire	SS1	197	75
Dandaru	SS2	143	87
Eleyele	SS3	192	72
Erinle	SS4	179	81

### Profile of antibiotic resistance phenotypes

In total, 315 confirmed *Vibrio* isolates were subjected to susceptibility test with 18 antibiotics belonging to 10 antimicrobial classes (supplementary Table 2). The antibiogram profile of the *Vibrio* isolates across the sampling sites varies. However, notable resistance to Sulphamethoxazole—83%, Tetracycline—70%, Erythromycin—82%, Rifampicin—80% were recorded in SS2 (Dandaru) whereas resistance to doxycycline were recorded in 72% *Vibrio* isolates in Erinle.

**Figure 3:**
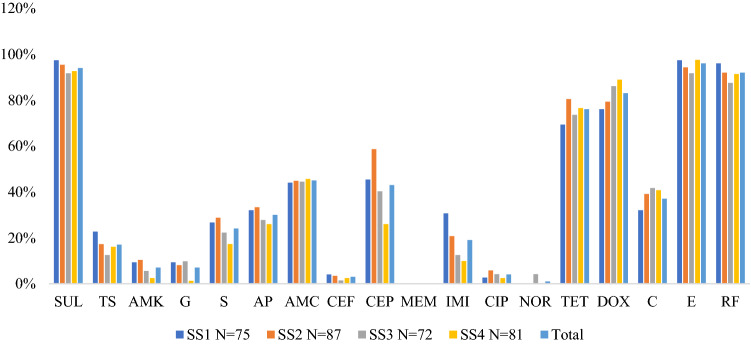
Antibiotic resistance profile of *Vibrio* isolates across the selected sampling sites. *CIP* ciprofloxacin, *AMK* amikacin, *MEM* meropenem, *E* erythromycin, *AMC* amoxicillin, *IMP* imipenem, *TS* trimethoprim–sulphamethoxazole, *CEF* cefotaxime, *C* chloramphenicol, *G* gentamicin, *SUL* sulphamethoxazole, *RF* rifampin, *CEP* cephalothin, *AP* ampicillin, *NOR* norfloxacin, *TET* tetracycline, *DOX* doxycycline, *S* Streptomycin.

None of the sample sites presented 100% susceptible or resistance of isolates to any classes of antibiotics. The total susceptibility of all *Vibrio* tested varied and follows the order: ciprofloxacin—93%, norfloxacin—98%, meropenem—91%, amikacin—76%, cefotaxime—88%, Trimethoprim + Sulphamethoxazole—75%, gentamicin—74%, ampicillin—54%. Although, high resistance to erythromycin—95%, sulfamethoxazole—94%, rifampicin—92%, doxycycline—82%, tetracycline—75% were observed as well as mild resistance to amoxicillin—45% and cephalothin—43% respectively (Fig. 3).

The antibiotic resistance and susceptibility characteristics across the sampling sites were analyzed by heatmap clusters (supplementary Fig. 1A–D). Two major clusters were formed column wise by the *Vibrio* isolates. The hetero-site isolates showed cluster characteristics indicating that all sampled rivers had 3 antibiogram clusters except river Eleyele with 4 hetero-site antibiogram clusters of the isolates.

### Phenotypes and indexes of multiple antibiotic resistance (MAR)

The multiple antibiotic resistance phenotype result indicated that all the isolates were resistance to three or more antibiotics except one (1) isolate at SS1 (Erinle).

MAR indexes of *Vibrio* isolates across the sampling sites ranged between 0.11 and 0.72 with MAR indexes less than 0.2 threshold limit obtained in SS2 (n = 2), SS3 (n = 4) and SS4 (n = 3). Highest (0.72) MAR index was recorded in SS3 with resistance to 13 of the tested antibiotics. Abundance of resistance pattern observed across the sample sites showed that SS1 had the lowest resistance pattern with ARPA of 0.106 while the remaining 3 sampled sites had 0.126 ARPA respectively. MAR phenotype pattern E-SUL-RF-TET-DOX with index of 0.38 was found to be the most common pattern to all the isolates across the sampling sites having being recorded in 8, 4,10 and 5 isolates in SS1 (Asejire), SS2 (Dandaru), SS3 (Eleyele) and SS4 (Erinle) respectively.

The MAR_index_ of 97% of *Vibrio*s from all the four rivers sampled were above 0.2 threshold value (Supplementary Table 3).

### Antibiotics resistance genes profile among *Vibrio* isolates

The profiling of antibiotic resistance genes was carried out on all resistance phenotypes of *Vibrio* isolates^25,26^. The profile of resistance genes of *Vibrio*s from the selected river water samples is as shown in Table 2. Among the 297 sulfonamide-resistant isolates assayed for resistance determinant genes, only 19% possessed the *sulI* gene, whereas 33% harbored *sulII* gene*.* However, 20 of the sulfonamide-resistant *Vibrio* isolates expressed dual *sulI-sulII* genes pattern (Table 3). Likewise, 237 tetracycline-resistant *Vibrio* isolates were examined for probable detection of three tetracycline resistance genes in which 28% were detected positive for *tetA* gene*,* 20% positive for *tetE* gene and 8% harbored *tet39* gene respectively. Also, dual and multiple resistant patterns were observed in the order: *tetA-tetE* (16), *tetE-tet39* (3), *tetA-tet39* (1) and *tetA-tetE-tet39* (1) (Table 3). Regarding the Beta-lactam antibiotics, the PCR amplification of 94-ampicillin-resistant *Vibrio* isolates revealed that 39% were *amp*C gene positive. Similarly, amoxicillin-resistant *Vibrio* isolates (141) were examined for probable detection of *bla*_*OXA*_ and *bla*_*PSE*_ genes. Both genes were spotted in 27% and 11% of the isolates respectively with dual *bla*_*PSE*_*-bla*_*OXA*_ resistant pattern observed in 8 of the isolates (Table 3).

**Table 2:** Incidence and distributions of antibiotics resistance genes among *Vibrio* isolates.

Antibiotics class	Antibiotics	Resistance gene	% of positive isolates per site				Total (%)
			SS1	SS2	SS3	SS4	
Sulfonamides	Sulphamethoxazole (n = 297)	*sul*I	18	13	9	15	55 (19)
		*sul*II	22	20	25	31	98 (33)
Beta-lactams	Ampicillin (n = 94)	*amp*C	13	11	4	9	37 (39)
	Amoxicillin (n = 141)	*bla*_*PSE*_	4	3	9	0	16 (11)
		*bla*_*OXA*_	2	11	7	5	25 (27)
Tetracyclines	Tetracyclines (n = 237)	*tet*A	23	15	18	9	66 (28)
		*tet*E	16	11	11	10	48 (20)
		*tet39*	3	2	3	0	8 (3)
Aminoglycosides	Gentamicin (n = 25)	aacC2	3	3	0	0	6 (24)
	Amikacin (n = 22)	*aphA1*	2	1	0	0	3 (14)
	Streptomycin (n = 75)	*str*A	10	9	4	6	29 (39)
Total			117	99	90	85	391

**Table 3:** Patterns of MAR-Determinants of *Vibrio* isolates.

Antibiotic family	Antibiotics	Multiple resistance-determinant pattern	Total
Sulfonamides	Sulphamethoxazole	*sulI–sulII*	20
Tetracyclines	Tetracycline	*tetA-tetE*	16
		*tetE-tet39*	3
		*tetA-tet39*	1
		*tetA-tetE-tet39*	1
Beta-lactams	Amoxicillin	*bla*_*PSE-*_*bla*_*OXA*_	8

**Figure 4:**
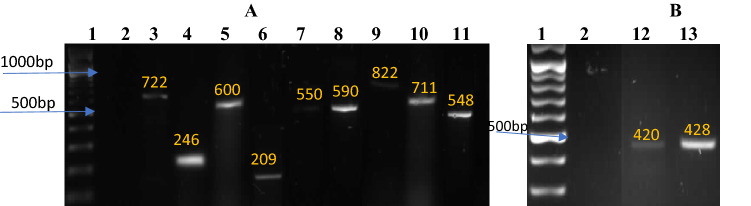
(**A**,**B**) Gel electrophoresis characteristic of antibiotics resistance genes in *Vibrio* Isolates. Lanes 1: Molecular marker (100 bp), lane 2: −ve control; lane 3: *sulII* (722 bp); lane 4: *tetE* (246 bp); lane 5: *aphA1* (600 bp); lane 6*: tetA* (209 bp); lane 7: *ampC* (550 bp); lane 8: *bla*_*OXA*_ (590); lane 9: *sulI* (822 bp); Lane 10: *tet39* (711 bp); lane 11: *strA* (548 bp); lane 12: *bla*_*PSE*_ (420 bp); lane 13: *aacC2* (428).

Among the 3 resistance genes encoding aminoglycosides, *aacC2* genes account for 24% of the 25 gentamicin-resistant *Vibrio* isolates whereas 14% of the amikacin-resistant isolates carried the *aphA1* gene while 39% of the streptomycin-resistant *Vibrio* isolates were identified to possess *strA* gene (Table 2). Overall, 391 resistance gene fingerprints were recovered across all sampled sites, with highest frequency recorded at SS1 (Asejire) with a total of 117 prints while SS4 had the lowest with 85 prints. Also, the occurrence of multiple antibiotics resistance genes ranged between 2 and 3 combinations with *tet* genes most occurring combinations followed by *sul* combination (Table 3). Generally, occurrence of resistance determinant follows the sequence: *sulII* > *tetA* > *sulI* > *tetE* > *ampC* > *strA* > *bla*_*OXA*_ > *bla*_*PSE*_ > *tet39* > *aacC2* > *aphA1*. The representative gel electrophoresis profile of antibiotic resistance determinant encoding the genes detected are as shown in Fig. 4 (A and B).

### Association between resistance determinants in *Vibrio* isolates

Statistical association was performed in order to determine probable relationship among resistance encrypting genes detected in *Vibrio* isolates and likewise to confirm if co-occurrence of a particular resistance genes could be established statistically. Significant associations were therefore established at *P* = *0.05.* the following positive associations were observed: *tetA* was more strongly associated with *sulI* and *tetE* but less strongly with *bla*_*OXA*_ and *bla*_*PSE*._ The *tetE* gene was also strongly associated *sul*II, *sul*I and *bla*_*OXA*_, but less strongly associated with *tet*39. Likewise, *strA* and *aacC2* showed strong association with *aphA1*, while *tet39* and *sulI* were less strongly associated with *bla*_*OXA*_ (Table 4). There was no negative correlation between any of the genes.

**Table 4:** Significance of *Vibrio* antibiotics resistance genes association^a^.

Gene	*tetA*	*tetE*	*tet39*	*Sul1*	*Sul11*	*bla*_*OXA*_	*bla*_*Pse*_	*ampC*	*strA*	*aacC*	*aphA1*
*aphA1tetA*	–										
*tetE*	++										
*tet39*	–	+									
*SulI*	+++	++	–								
*SulII*	–	++	–	–							
*bla*_*OXA*_	+	++	+	+	–						
*bla*_*Pse*_	+	–	–	–	–	–					
*ampC*	–	–	–	–	–	–	+				
*strB*	–	–	–	–	–	–	–	–			
*aacC*	–	–	–	–	–	–	–	+	+		
*aphA1*	–	–	–	–	–	–	–	–	++	++	–

^a^Only antibiotic resistance genes with a P 0.05 level of connection with another gene are shown. The level of significance was set at: P 0.05; +, P 0.01; ++, P 0.01; and +++, P 0.001 (as determined by the chi-square exact test).

## Discussion

Pollution of water bodies also influence the microbial population and species of organisms found in them. *Vibrio* species are freshwater-associated organisms implicated in both human and animal infections. In this study, the incidence of *Vibrio* species was examined and 58% of the presumptive *Vibrio* spp. were confirmed after molecular confirmation, with Eleyele river (SS3) and Dandaru river (SS2) exhibiting the highest prevalence. Occurrence of high *Vibrio* prevalence in Erinle river suggested the presence of healthy *Vibrio* carriers who shed *Vibrio* into the environment on a regular basis. Although severe rainfall modifies the physical and chemical parameters in the aquatic environment, the subsequent influx of flood water together with organic waste (such as food, sewage, fertilizer and green waste as well as human and animal feces.) can aid *Vibrio* species proliferation in the chosen rivers. This finding is consistent with earlier studies that recorded high incidence of *Vibrio* species^27,28^. Furthermore, prior research has confirmed the link between Vibrio species and the aquatic environment^6,25^. The incidence of *Vibrio* spp. did not differ significantly between the sampled sites, according to statistical analysis.

Water ecosystems have become a well-known hotspot for environmental diffusion of both *Vibrio* species and antimicrobials^29^. Although, *Vibrios* are largely considered to be very susceptible to most antimicrobials recommended for medical treatment^30^, resistance among environmental isolates of *Vibrio* is increasingly being recorded in many countries.

Eighteen antibiotics from 9 different classes were examined in this investigation. Multiple antibiotics resistance was observed in the recovered *Vibrio* isolates against some endorsed and frequently used antibiotics in *Vibrio-*associated infection treatment which correlated with previous reports^31^. Remarkable resistance against erythromycin and sulfamethoxazole observed in this study is comparable with previous reports on clinical and environmental *Vibrio* isolates^32,33^. Likewise, relative resistance against the two tested β-lactams drugs (Amoxicillin 45% and amikacin 30%) recorded in this study is similar to that documented in environmental *Vibrio* isolates elsewhere^34–36^. The observed resistance of *Vibrios* to tested β-lactams in this study maybe related with the isolation source as bacteria such as *Vibrio,* recovered from water environment have been documented to show high resistance towards β-lactams class of antibiotics^37,38^. The resistance to amoxicillin and amikacin observed in this study is not surprising as these drugs enjoys widespread usage across different settings. Moreover, the frequency of antibiotics use over time has been hypothesized by researchers to contribute to development of resistance to antibiotics among bacteria^36,39^. Resistance to imipenem (18%) was also recorded in this study which corresponds to studies on *Vibrio* spp. recovered from shellfish and fish in Egypt^33^ and marine environment in Norway^40^. The recorded resistance to imipenem in this present study raises public health concerns as this drug is one of the major drugs of last resort for medical treatment of multiple drug-resistance *Vibrio* infections. Moreso, carbapenemases responsible for resistance to carbapenems has been associated with mobile genetic elements which can perhaps be transferred to humans through food interface^41^. Resistance recorded against tetracycline and cephalothin showed comparable profile with those stated in various studies across other countries^42–44^ and signifies public health threat as these are prescribed drugs in the treatment of humans infections^42,45^. High susceptibility to norfloxacin (98%), ciprofloxacin (93%), meropenem (91%), cefotaxime (88%), amikacin (76%), trimethoprim + sulfamethoxazole (75%), gentamicin (74%) recorded in this study is comparable to studies elsewhere^46^. Generally, varied resistance among antibiotics belonging to the sulfonamides, β-lactams, aminoglycosides, macrolides and tetracycline classes obtained in this study is similar to other findings^22,35,47^ in which similar findings of multiple antibiotic resistance of *Vibrio* spp. recovered from aquatic habitat have also been reported.

The high level of *Vibrio* isolate resistance to many of the antibiotics examined across all sample sites in this present study suggests a probable misuse or overuse of antimicrobials around the study area for a variety of purposes beyond what the environment can absorb, thereby resulting in the maintenance of unabsorbable residue in the water bodies. Such practices have been suggested to impact the likelihood of bacteria to develop resistance to commonly used antibiotics^26^. Furthermore, it has been hypothesized that the ability of microbes to share their genetic material in fresh and aquatic environments is a contributing factor to the rise in antibiotic resistance^48^. In light of the possible influence on treatment outcomes, the continued development of antimicrobial drug resistance to the standard oral treatments for *Vibrio*-associated diseases raises public health concerns^6^.

The degree of exposure of all the four rivers to contamination by antibiotics and its possible human health risk was assessed by the multiple antibiotic resistance index (MARI) which shows that the indexes range from 0.06 to 0.72 across all the sampled sites.

MARI of 0.06, 0.11 and 0.16 recorded in few of the *Vibrio* isolates recovered from SS2 (n = 2), SS3 (n = 4) and SS4 (n = 3) indicates low risk of environmental contamination. However, higher MARI values obtained across the sampling sites with highest (0.72) recorded in SS3 having exhibited resistance to 13 of the examined antibiotics indicates the *Vibrio* isolates have a high ability to contaminate the water sources; this is in accord with results documented in other findings^25,42,43^. MAR phenotype pattern E-SMX-RP-T-DXT with index of 0.38 was found to be the most common pattern among the isolates concurs with a study on *Vibrio* parahaemolyticus from shellfish in Selangor Malaysia in which resistance to five antibiotics was found prevalent among the isolates^49^. it was observed in this study that MAR_index_ of 98% of *Vibrio* isolates from all the four rivers sampled greater than the threshold value. Similar findings of high MAR index of *Vibrio* spp. has been reported in aquatic environment elsewhere^50–52^. The high multiple resistance observed signifies a possible treat public health in the area of bacterial infection control and managements as these antibiotics are still the drug of choice in managing *Vibrio* related infection in both human and animal. According to many studies, the role of wastewater run-off in diffusion of antibiotic resistance in intestinal bacteria harboring resistant plasmids cannot be over-emphasized^53^.

Different samples recovered from environmental sources have been found to harbor diverse antimicrobial genes that encodes resistance to antibiotics such as aminoglycosides, β-lactams, tetracycline, chloramphenicol sulfonamides and trimethoprim^25,48,54^. In this present study, 11 different resistant genes were assayed on phenotypically resistant *Vibrio* isolates. Among the sulfonamide and tetracycline resistance genes tested, *tet*A (28%) and *sul*II (33%) had the highest frequency of occurrence. This outcome is similar to reports by^55^ in which *tetA* (64.28%) was among the highest detected genes in *Vibrio* parahaemolyticus isolated from Malaysian seawater and fishes^56^ and with high detection of *sul*II (53.8%) genes in *Vibrio cholerae* recovered from Hilsha fish.

Specifically, *sul*I gene is linked with class1 integron while *sul*ll are typically found on large infectious multi-resistant or small non-conjugative plasmids which may contribute to the commonness of *sul*ll detection in water environment. The detection of *tet*A and *sul*II genes advocates that they are extensively used antibiotics in animal and human therapy in this region. This is because tetracyclines and sulfonamides have broad spectrum of activities and are relatively cheap^57^. They are commonly incorporated into livestock feed as growth promoters in aquaculture. Likewise, the two classes of antibiotics are used in treatment of *Vibrio* associated infections in humans, as sulfonamide drugs are used as combination drug for cholera treatment in both children and adults^57,58^. This findings match reports of other studies where *sul*II gene detection was reported as the most profuse sulfonamide resistance conferring gene in freshwater environments^25,59–62^. Likewise, high *tet*A genes prevalence is consistent with^63,64^ reports in which the ribosomal efflux gene, *tet*A, have been found to be the most commonly detected resistance genes in *Vibrio* spp. from fresh environment attesting that t*et*A gene can be easily harbored by different class of bacteria due to their affinity for diverse host range.

Beta-lactams, an extensively used antibiotics in the management of several infections are usually distributed in freshwater environment and consequently pose substantial risk to environmental health. Three β-lactams genes *bla*_*OXA,*_* bla*_*PSE*_ and *amp*C were examined. However, *amp*C had the highest frequency of recovery among all tested isolates. As stated in earlier studies, diverse pools of β-lactamases are existing in many environmental isolates of *Vibrio* species signifying that such acquirement of β-lactamase genes is not primarily due to β-lactamase treatment's selective pressure^65^. Some gram-negative environmental isolates have been found to naturally have low levels of β-lactam antibiotic resistance. It has been stated also that some intrinsic *amp*C resistance gene found in Enterobacteriaceae are positioned on mobile genetic element with the ability to transmit between diverse bacteria. Some other studies have also recorded high prevalence of *amp*C gene in gram negative bacteria from freshwater environment^66^.

Aminoglycosides are a type of antibiotic that has become increasingly significant in the management and prevention of severe bacterial infections in both animals and humans since resistance to diverse classes of antibiotics are increasing in gram negative bacteria. Among the different aminoglycosides genes that were examined, the *str* genes that bestows streptomycin phosphoryl transferase resistance, had more *str*A prevalence than others. These findings agree with similar studies in which high occurrence of *str*A detection were reported in aquatic bacteria^67,68^. Several means of acquiring resistance to aminoglycosides has been described; modification of enzymes such as neomycin phosphor-transferase, alteration of chromosome binding sites and altered aminoglycoside uptake/efflux pumps which bestows cross- resistance and change in cell membrane.

The correlation between the emergence of aminoglycoside resistance and the widespread use of antibiotics for cholera prevention and treatment as well as other usage is well known. The expression of multiple drug resistant by non-cholera *Vibrio* isolates to some of the antibiotics conventionally used in treatment of cholera calls for concern as this could have direct influence on the treatment of cholera cases in Southwestern Nigeria and other parts of the country where the organism could spread.

This study reiterated that antibiotic susceptibility and PCR assays are complementary. The PCR assay shows the existence of genes linked with resistance in many of the phenotypically resistant strains and absence of resistance conferring genes in some other strains that shows phenotypic resistance. As observed, *tet*A and *sul*II had the highest frequency of genotypic resistance occurrence while erythromycin, rifampin and sulphamethoxazole showed the most prevalent phenotypic resistance. Reasons for the observed discrepancy between genotypic and the phenotypic antibiotic resistance might be presence of expression profiles that were not subjected to testing due to limitations in selecting genes in this study as reported elsewhere^69^. Also, there is possibility that some of the ARGs in these *Vibrios* are unexpressed genes^61^. Consequently, these genes might be possible source of risk in the environment because apart from contributing to the resistance reservoir, they could also be horizontally transferred under suitable conditions. This suggests complexity of the linkage between phenotypes and genetic constructions. The results obtained were put through a statistical test to see if there was any correlation between the various resistance genes investigated, and high correlations were found among some of the resistance determinants. Notable comparable association between the resistance genes indicates *tet*A was strongly associated with *sul*I and both genes were the most detected among the three tetracycline and sulfonamide resistance genes tested. High proliferation of *sul*II than *sul*I genes in freshwater bacterial isolates is well documented^26,70^. This observation further affirms that resistance to antibiotics is triggered by more than two genes.

Antibiotic resistance development and persistent in the freshwater environment has been established to be initiated by various resistance mechanism such as selective pressure of antimicrobial substances^71^ horizontal gene transfer predispositions for genetic mutations, recombination events^66^ and the dissemination of antibiotic-resistant bacteria from human and veterinary medicine^72^. These mechanisms assist microorganisms to acclimatize to varying environments and contribute to the widely reported antibiotic resistance patterns observed in aquatic habitats. Furthermore, the genotypic resistance information can be conveyed via mobile genetic elements (plasmid, integron, transposon) from a particular species to different species (horizontal gene transfer)^73,74^. Also, antibiotics in the aquatic environment can lead to transformation in the ecological roles of microbes^75^ apart from encouraging the development and spreading of antibiotic resistant microorganisms.

## Conclusion

The high concentrations of antibiotic resistant organisms and antibiotic resistant genes in the rivers examined indicated the rivers are polluted and highly impacted by waste discharge and other anthropogenic activities around the rivers. It is anticipated that unregulated waste disposal into the rivers and usage of antimicrobials in agriculture, clinical and domestic setting will keep on generating pools of resistance genes within the surface waters. Also, this can create a selective pressure in the immediate environment that would lead to genetic mutations in both allochthonous and autochthonous microorganisms thereby enabling them to persistently thrive and multiply. These observations therefore buttress the importance of environmental context-tailored strategies for continuous monitoring and surveillance of the aquatic environments for the presence of emerging contaminants such as, antibiotic resistance bacteria and their resistance determinant in order to evaluate their potential effects on environmental and human health.

## Supplementary Information

Supplementary Information.

## Data Availability

All data are listed in the main manuscript and other associated data are listed supplementary file.
