# Supplementary material for: Incidence of antibiotic resistance genotypes of Vibrio species recovered from selected freshwaters in Southwest Nigeria
Source: Sci Rep. 2022 Nov 7;12:18912. doi: 10.1038/s41598-022-23479-0 (PMC9640555; doi:10.1038/s41598-022-23479-0)
Supplement: Supplementary file 1 — Supplementary Information. [file 41598_2022_23479_MOESM1_ESM.docx]

**Table 1: Amplification condition for resistance genes**

| Sulfonamides | *sul1* | F: TCGGCATTCTGAATCTCAC  R: TGATCTAACCCTCGGTCTC | 822 | Initial Denaturation at 94 °C for 5 min,  Then 35 cycles of:  denaturation - 94 °C for 1 min  annealing - 55 °C for 1 min,  extension -72 °C for 5 min, followed by  final extension 72 °C for 5 min | Maynard *et al*., 2004 |
| --- | --- | --- | --- | --- | --- |
|  | *sul11* | F: GGCATCGTCAACATAACC  R: GTGTGCGGATGAAGTCAG | 722 | Initial Denaturation at 94 °C for 5 min,  Then 30 cycles of:  denaturation - 94 °C for 30 s  annealing - 50 °C for 30 s,  extension -72 °C for 1.5 min, followed by  final extension 72 °C for 5 min | Falbo *et al.*, 1999 |
| Tetracyclines | *tetA* | F: GCTACATCCTGCTTGCCTTC  R: ATAGATCGCCGTGAAGAGG | 209 | Initial Denaturation at 94 °C for 5 min,  Then 35 cycles of:  denaturation - 94 °C for 1 min  annealing - 55 °C for 1 min,  extension - 72 °C for 1.5 min, followed by  final extension - 72 °C for 5 min | Ng *et al*., 2001 |
|  | *tetE* | F: GCGCTNTATGCGTTGATGCA  R: ATGTGTCCTGGATTCCT | 246 | Initial Denaturation at 94 °C for 5 min,  Then 30 cycles of:  denaturation - 94 °C for 30 s  annealing - 55 °C for 30 s,  extension - 72 °C for 30 s, followed by  final extension - 72 °C for 5 min | Jun *et al*., 2004 |
|  | *tet39* | F: CTCCTTCTCTATTGTGGCTA  R: CACTAATACCTCTGGACATCA | 711 | Initial Denaturation at 94 °C for 5 min,  Then 30 cycles of:  denaturation - 94 °C for 30 s  annealing - 55 °C for 30 s,  extension -72 °C for 30 s, followed by  final extension - 72 °C for 5 min | Agerso and Peterson, 2007 |
| Aminoglycosides | *strA* | F: CTTGGTGATAACGGCAATTC  R: CCAATCGCAGATAGAAGGC | 548 | Initial Denaturation at 94 °C for 4 min,  Then 30 cycles of:  denaturation - 94 °C for 45 s  annealing - 50 °C for 45 s,  extension - 72 °C for 1.5 min, followed by  final extension - 72 °C for 5 min | Velusamy *et al.,* 2007 |
|  | *apHa2* | F: GAACAAGATGGATTGCACGC  R: GATGTTTCGCTTGGTGGTC | 412 | Initial Denaturation at 94 °C for 4 min,  Then 30 cycles of:  denaturation - 95 °C for 30 s  annealing - 55 °C for 45 s,  extension -72 °C for 1 min, followed by  final extension 72 °C for 7 min | Zhu, 2007 |
|  | *aacC2* | F: CGGAAGGCAATAACGGAG  R: TCGAACAGGTAGCACTGAG | 428 | Initial Denaturation at 94 °C for 5 min,  Then 30 cycles of:  denaturation - 94 °C for 30 s  annealing - 50 °C for 30 s,  extension -72 °C for 1.5min, followed by  final extension - 72 °C for 5 min | Maynard *et al.,* 2004 |
| Beta-lactams | *ampC* | F: TTCTATCAAMACTGGCARCC  R: CCYTTTTATGTACCCAYGA | 550 | Initial Denaturation at 94 °C for 4 min,  Then 30 cycles of:  denaturation - 94 °C for 45 s  annealing - 60 °C for 45 s,  extension -72 °C for 45 s, followed by  final extension - 72 °C for 7 min | Velusamy *et al.,* 2007 |
|  | *bla_OXA_* | F: ACCAGATTCAACTTTCAA R: TCTTGGCTTTTATGCTTG | 590 | Initial Denaturation at 94 °C for 3 min,  Then 30 cycles of:  denaturation - 94 °C for 1 min  annealing - 58°C for 1 min,  extension -72 °C for 1 min, followed by  final extension - 72 °C for 7 min | Letchumanan *et al.,* 2015 |
|  | *bla_PSE_* | F: CGCTTCCCGTTAACAAGTAC  R: CTGGTTCATTTCAGATAGCG | 420 | Initial Denaturation at 94 °C for 5 min,  Then 30 cycles of:  denaturation - 94 °C for 30 s  annealing - 55 °C for 30 s,  extension -72 °C for 1 min, followed by  final extension - 72 °C for 7 min | Kim *et al*., 2013 |

| **Susceptible (S) per**  **Sample sites** |  | **Resistance (R) per**  **sample sites** |  | **Intermediate**  **(I) TOTAL** |
| --- | --- | --- | --- | --- |

**TABLE 2: Antibiogram profile of *Vibrio* isolates across the selected sampling site**

| Antimicrobial family | Antimicrobial agent | Disc code | Potency (µg) | SS1  N=75 | SS2  N=87 | SS3 N=72 | SS4  N=81 | **Total**  **S (%)** | SS1  N=75 | SS2  N=87 | SS3 N=72 | SS4  N=81 | **Total**  **R (%)** | **Total**  **I (%)** |
| --- | --- | --- | --- | --- | --- | --- | --- | --- | --- | --- | --- | --- | --- | --- |
|  | Sulphamethoxazole | SUL | 25 | 0 | 2 | 0 | 2 | **4(1.27)** | 73 | 83 | 66 | 75 | **297(94.2)** | **14(4.44)** |
|  | Trimethoprim +  Sulphamethoxazole | TS | 25 | 55 | 61 | 58 | 61 | **235(74.60)** | 17 | 15 | 9 | 13 | **54(17.14)** | **26(8.25)** |
| Aminoglycosides | Amikacin | AMK | 30 | 56 | 56 | 59 | 67 | **238(75.56)** | 7 | 9 | 4 | 2 | **22(6.98)** | **55(17.46)** |
|  | Gentamicin | G | 10 | 54 | 62 | 50 | 67 | **233(73.97)** | 7 | 7 | 7 | 1 | **22(6.98)** | **60(19.05)** |
|  | Streptomycin | S | 10 | 31 | 31 | 32 | 46 | **130(41.27)** | 20 | 25 | 16 | 14 | **75(23.81)** | **110(34.92)** |
| β-lactams | Ampicillin | AP | 10 | 34 | 40 | 46 | 50 | **170(53.97)** | 24 | 29 | 20 | 21 | **94(29.84)** | **51(16.19)** |
|  | Amoxycillin | AMC | 25 | 27 | 33 | 35 | 30 | **125(39.68)** | 33 | 39 | 32 | 37 | **141(44.76)** | **49(15.56)** |
| Cephems | Cefotaxime | CEF | 30 | 62 | 72 | 68 | 77 | **279(88.57)** | 3 | 3 | 1 | 2 | **9(2.86)** | **27(8.57)** |
|  | Cephalothin | CEP | 30 | 36 | 31 | 41 | 50 | **158(50.16)** | 34 | 51 | 29 | 21 | **135(42.86)** | **22(6.98)** |
| Carbapenems | Meropenem | MEM | 10 | 61 | 80 | 68 | 78 | **287(91.11)** | 0 | 0 | 0 | 0 | **0(0)** | **28(8.89)** |
|  | Imipenem | IMI | 10 | 32 | 36 | 41 | 48 | **157(49.84)** | 23 | 18 | 9 | 8 | **58(18.41)** | **100(31.75)** |
| Fluoroquinolones | Ciprofloxacin | CIP | 5 | 71 | 81 | 64 | 77 | **293(93.01)** | 2 | 5 | 3 | 2 | **12(3.81)** | **10(3.17)** |
|  | Norfloxacin | NOR | 30 | 73 | 86 | 69 | 80 | **308(97.78)** | 0 | 0 | 3 | 0 | **3(0.95)** | **4(1.27)** |
| Tetracycline | Tetracycline | TET | 30 | 20 | 13 | 17 | 17 | **67(21.27)** | 52 | 70 | 53 | 62 | **237(75.24)** | **11(3.49)** |
|  | Doxycycline | DOX | 30 | 10 | 8 | 7 | 12 | **37(11.75)** | 57 | 69 | 62 | 72 | **260(82.54)** | **18(5.71)** |
| Phenicol | Chloramphenicol | C | 30 | 28 | 29 | 23 | 41 | **121(38.41)** | 24 | 34 | 30 | 33 | **116(36.83)** | **78(24.76)** |
| Macrolides | Erythromycin | E | 15 | 0 | 4 | 4 | 5 | **13(4.13)** | 73 | 82 | 66 | 79 | **300(95.24)** | **2(0.63)** |
| Rifamycin | Rifampin | RF | 5 | 3 | 4 | 3 | 3 | **13(4.13)** | 72 | 80 | 63 | 74 | **289(91.75)** | **13(4.13)** |

**Table 3: Patterns, indexes and abundance of MAR phenotypes**

| **No of antimicrobials** | **Resistance pattern** | **No Observed** | MARI | | ARPA |
| --- | --- | --- | --- | --- | --- |
|  | **Sampling site=SS1(N=75)** |  | |  | 0.106 |
| 4 | E-SUL-RF-CEP  E-RF-TET-DOX  E-TS-SUL-RF  E-SUL-RF-AP | 3  1  1  1 | | 0.22  0.22  0.22  0.22 |  |
| 5 | E-SUL-RF-TET-D0X | 8 | | 0.27 |  |
|  | E-AMC-RF-TET-DOX  E-AMC-SUL-RF-DOX  E-SUL-RF-AP-S  E-AMC-SUL-RF-CEP | 1  1  1  1 | | 0.27  0.27  0.27  0.27 |  |
| 6 | E-C-SUL-RF-CEP-DOX  E-AMC-SUL-RP-TET-DOX  E-SUL-RF-AP-TET-DOX  E-SUL-RF-CEP-TET-DOX  E-TS-SUL-RF-TET-DOX  E-AMC-TS-SUL-CEP-S  E-AMC-SUL-RF-AP-CEP  E-SUL-RF-AP-CEP-DOX  AMK-AMC-TS-C-SUL-S | 2  4  2  1  1  1  1  1  1 | | 0.33  0.33  0.33  0.33  0.33  0.33  0.33  0.33  0.33 |  |
| 7 | E-AMC-IMI-SUL-RF-AP-CEP  E-AMC-TS-SUL-RF-TET-DOX  E-IMI-SUL-RF-CEP-TET-DOX  E-IMI-SUL-RF-AP-TET-DOX  E-AMK-IMI-SUL-RF-TET-DOX  E-C-SUL-RF-CEP-TET-DOX  E-AMC-GM-SUL-RF-TET-DOX  E-AMC-SUL-RF-CEP-TET-DOX  E-AMC-C-SUL-RF-CEP-DOX  E-SUL-RF-AP-TET-S-DOX  TS-C-SUL-RF-TET-S-DOX  E-AMC-TS-C-SUL-RF-DOX  E-CIP-TS-C-SUL-RF-TET | 2  2  1  1  1  1  1  1  1  1  1  1  1 | | 0.38  0.38  0.38  0.38  0.38  0.38  0.38  0.38  0.38  0.38  0.38  0.38  0.38 |  |
| 8 | E-IMI-C-SUL-RF-AP-CEP-S  E-IMI-GM-C-SUL-RF-AP-CEP  E-IMI-GM-SUL-RF-TET-S-DOX  E-IMI-SUL-RF-CEP-TET-S-DOX  E-TS-IMI-SUL-RF-TET-S-DOX  E-AMK-IMI-C-SUL-RF-TET-DOX  E-AMK-IMI-SUL-RF-AP- TET-DOX  E-AMK-IMI-SUL-AP-TET-S-DOX  E-AMC-C-SUL-RF-CEP-TET-DOX  E-AMC-SUL-RF-AP-CEP-TET-DOX  E-AMC-TS-C-SUL-RF-TET-S | 2  1  1  1  1  1  1  1  1  1  1 | | 0.44  0.44  0.44  0.44  0.44  0.44  0.44  0.44  0.44  0.44  0.44 |  |
| 9 | E-AMC-TS-IMI-SUL-RF-CEP-TET-DOX  E-AMK-AMC-IMI-SUL-RF-CEP-TET-DOX  E-AMC-IMI-C-SUL-RF-CEP-TET-DOX  E-AMC-GM-SUL-RF-AP-CEP-TET-DOX  E-AMC-C-SUL-RF-AP-CEP-S-DOX  E-CIP-TS-GM-C-SUL-TET-S-DOX  E-AMC-C-SUL-RF-AP- CEP-TET-DOX  E-AMC-CEF-C-SUL-RF-AP-CEF-TET | 3  1  1  1  1  1  1  1 | | 0.50  0.50  0.50  0.50  0.50  0.50  0.50 |  |
| 10 | E-AMC-IMI-C-SUL-RF-AP-TET-S-DOX  E-AMK-IMI-GM-SUL-RF-AP-TET-S-DOX  E-AMC-IMI-C-SUL-RF-AP-CEP-S-DOX  E-TS-CEF-C-SUL-RF-AP-CEP-TET-DXT | 1  1  1  1 | | 0.55  0.55  0.55  0.55 |  |
| 11 | E-TS-CEF-GM-C-SUL-RF-CEP-TET-S-DOX  E-AMC-IMI-C-SUL-RF-AP-CEP-TET-S-DOX | 1  1 | | 0.61  0.61 |  |
|  | **Sampling site=SS2 (N=87)** |  | | 0.126 |  |
| 2 | CEF-SUL | 1 | | 0.11 |  |
| 3 | E-RF-TET | 1 | | 0.16 |  |
| 4 | E-RF-TET-DOX | 1 | | 0.22 |  |
|  | SUL-RF-AP-TET  E-AMC-TET-DOX  E-TS-C-SUL  E -SUL-RF-DOX | 1  1  1 | | 0.22  0.22  0.22 |  |
| 5 | E-SUL-RF-CEP-TET  E-SUL-RF-TET-DOX  E-AMC-RF-TET-DOX  E-SUL-RF-S-DOX  E-AMC-CEF-C-SUL | 2  4  1  1  1 | | 0.27  0.27  0.27  0.27  0.27 |  |
| 6 | E-C-SUL-RF-TET-DOX  E- AMC-SUL-TET-S-DOX  E-GM-SUL-RF-TET-DOX  E- AMK-SUL-RF-TET-DOX  E- AMC-SUL-RF-TET-DOX  E- TS-SUL-RF-TET-DOX  E-SUL-RF-AP-CEP-DOX  E-IMI-SUL-RF-AP-CEP  TS-C-SUL-RF-TET-S  E-SUL-RF-CEP-TET-DOX | 3  1  2  1  3  1  1  2  1  1 | | 0.33  0.33  0.33  0.33  0.33  0.33  0.33  0.33  0.33  0.33 |  |
| 7 | E-SUL-RF-AP-CEF-TET-DOX  E-AMC-IMI-SUL-RP-CEP-DOX  E-IMI-C-SUL-RF-TET-DOX  E-AMC-IMI-GM-SUL-RF-CEP  E-SUL-RF-CEP-TET-S-DOX  E-AMK-SUL-RF-CEP-TET-DOX  E-AMC-SUL-RF-CEP-TET-DOX  E-IMI-C-SUL-RF-CEP-DOX  E-AMC-C-SUL-RF-TET-DOX  E-AMC-TS-SUL-RF-CEP-S  E-AMC-SUL-RF-AP-CEP-S  AMC-SUL-RF-AP-CEP-TET-DOX  E-AMC-TS-SUL-TET-S-DOX  E-AMC-SUL-RF-CEP-TET-DOX | 1  1  2  1  2  1  4  1  1  1  1  1  1  1 | | 0.38  0.38  0.38  0.38  0.38  0.38  0.38  0.38  0.38  0.38  0.38  0.38  0.38  0.38 |  |
| 8 | E-AMC-IMI-SUL-RF-CEP-TET-DOX  E-AMC-IMI-SUL-RF-CEP-TET-S  E-AMC-SUL-RF-AP-CEP-TET-DOX  E-AMC-IMI-SUL-RF-TET-S-DOX  E-AMC-TS-SUL-RF-CEP-TET-DOX  E-AMK-CIP-C-SUL-RF-TET-DOX  E-CIP-SUL-RF-AP-CEP-TET-DOX  E-AMC-IMI-SUL-RF-CEP-TET-S  E-C-SUL-RF-AP-CEP-TET-DOX  E-AMK-IMI-C-SUL-RF-AP-CEP | 1  1  1  1  1  1  1  1  2  1 | | 0.44  0.44  0.44  0.44  0.44  0.44  0.44  0.44  0.44  0.44 |  |
| 9 | E-AMC-IMI-SUL-RF-AP-CEF-TET-DOX  E-AMK-IMI-C-SUL-RF-TET-S-DOX  E-AMC-C-SUL-RF-AP-CEP-TET-DOX  E-TS-IMI-C-SUL-RF-TET-S-DOX  E-IMI-GM-C-SUL-RF-TET-S-DOX  E-AMK-AMC-TS-SUL-RF-CEP-TET-DOX  E-AMC-C-SUL-RF-CEP-TET-S-DOX  E-GM-C-SUL-RF-AP-CEP-TET-DOX  E-AMK-AMC-IMI-SUL-RF-AP-CEP-S  E-CEF-C-SUL-RF-AP-CEP-TET-DOX  E-AMC-SUL-RF-AP-CEP-TET-S-DOX  E-C-SUL-RF-AP-CEP-TET-S-DOX | 1  1  4  1  1  1  2  1  1  1  1  1 | | 0.50  0.50  0.50  0.50  0.50  0.50  0.50  0.50  0.50  0.50  0.50  0.50 |  |
| 10 | E-AMC-IMI-C-SUL-RF-AP-CEP-TET-DOX  E-CIP-AMC-TS-SUL-RF-CEP-TET-S-DOX  E-CIP-TS-C-SUL-RF-CEP-TET-S-DOX  E-TS-C-SUL-RF-AP-CEP-TET-S-DOX  E-AMK-AMC-TS-SUL-RF-AP-CEP-TET-DOX  E-AMC-TS-GM-C-SUL-RF-AP-CEP-S | 1  1  1  1  1  1 | | 0.55  0.55  0.55  0.55  0.55  0.55 |  |
| 11 | E-AMC-TS-C-SUL-RF-AP-CEP-TET-S-DOX  E-CIP-TS-C-SUL-RF-AP-CEP-TET-S-DOX | 1 | | 0.61  0.61 |  |
| 12 | E-AMC-IMI-GM-C-SUL-RF-AP-CEP-TET-S-DOX | 1 | | 0.66 |  |
|  | **Sampling site=SS3 (N=72)** |  | | 0.126 |  |
| 3 | E-SUL-RF  E-TET-DOX  SUL-RF-S | 2  1  1 | | 0.16  0.16  0.16 |  |
| 4 | E-RF-TET-DOX  E-SUL-TET-DOX | 2  1 | | 0.22  0.22 |  |
| 5 | E-SUL-RF-TET-DOX  E-SUL-RF-CEP-DOX  E-AMC-RF-TET-DOX  E-AMC-SUL-TET-DOX  AMC-SUL-RF-AP-TET  E-SUL-RF-AP-CEP | 10  1  1  1  1  1 | | 0.27  0.27  0.27  0.27  0.27  0.27 |  |
| 6 | E-C-SUL-RF-TET-DOX  E-AMC-SUL-RF-CEP-DOX  E-IMI-SUL-RF-TET-DOX  E-AMC-SUL-RF-TET-DOX  E-SUL-RF-TET-S-DOX  E-SUL-RF-CEP-TET-DOX  E-C-SUL-RF-CEP-DOX  E-AMC-C-SUL-RF-CEP  E-TS-C-SUL-TET-DOX | 4  1  1  6  1  1  1  1  1 | | 0.33  0.33  0.33  0.33  0.33  0.33  0.33  0.33  0.33 |  |
| 7 | E-IMI-SUL-RF-CEP-TET-DOX  E-AMC-SUL-RF-CEP-TET-DOX  E-C-SUL-RF-AP-CEP-DOX  E-AMK-AMC-SUL-RF-TET-DOX  E-C-SUL-RF-CEP-TET-DOX  E-AMC-SUL-RF-AP-CEP-DOX  AMK-TS-GM-C-SUL-NOR-S  AMC-TS-GM-C-SUL-NOR-S  E-C-SUL-RF-TET-S-DOX  E-C-SUL-AP-CEP-TET-DOX  E-AMC-C-SUL-RF-TET-DOX | 1  3  2  1  1  2  1  1  1  1  1 | | 0.38  0.38  0.38  0.38  0.38  0.38  0.38  0.38  0.38  0.38  0.38 |  |
| 8 | E-AMC-IMI-SUL-RF-CEP-TET-DOX  E-IMI-C-SUL-RF-TET-S-DOX  E-AMC-C-RF-AP-TET-S-DOX  E-AMC-C-SUL-RF-AP-CEP-DOX  E-AMC-TS-SUL-RF-AP-CEP-S | 1  1  1  1  1 | | 0.44  0.44  0.44  0.44  0.44 |  |
| 9 | E-AMC-C-SUL-RF-AP-CEP-TET-DOX  E-AMC-TS-C-SUL-RF-TET-S-DOX  AMK-CIP-GM-C-SUL-RF-CEP-NOR-S | 2  1  1 | | 0.50  0.50  0.50 |  |
| 10 | E-TS-C-SUL-RF-AP-CEP-TET-S-DOX  E-CIP-AMC-SUL-RF-AP-CEP-TET-S-DOX | 1  1 | | 0.55  0.55 |  |
| 11 | E-AMC-TS-IMI-C-SUL-RF-AP-TET-S-DOX  E-AMC-IMI-GM-C-SUL-AP-CEP-TET-S-DOX  E-AMC-IMI-GM-C-SUL-RF-AP-CEP-TET-DOX | 1  1  1 | | 0.61  0.61  0.61 |  |
| 12 | E-AMC-IMI-GM-C-SUL-RF-AP-CEP-TET-S-DOX  E-CIP-AMC-TS-GM-C-SUL-RF-AP-CEP-TET-DOX | 1  1 | | 0.66  0.66 |  |
| 13 | E-AMK-TS-IMI-CEF-C-SUL-RF-AP-CEP-TET-S-DOX | 1 | | 0.72 |  |
|  | **Sampling site=SS4 (N=81)** |  | | 0.126 |  |
| 1 | DOX | 1 | | 0.06 |  |
| 2 | AMC-S | 1 | | 0.11 |  |
| 3 | E-SUL-RF | 1 | | 0.16 |  |
| 4 | E-SUL-RF-CEP  E-RF-TET-DOX  E-SUL-RF-DOX | 1  1  1 | | 0.22  0.22  0.22 |  |
| 5 | E-SUL-RF-CEP-DOX  E-SUL-RF-TET-DOX  E-RF-CEP-TET-DOX  E-AMC-SUL-RF-CEP  E-TS-SUL-RF-CEP  E-TS-SUL-TET-DOX  E-AMC-C-SUL-DOX | 1  5  1  1  1  1  1 | | 0.27  0.27  0.27  0.27  0.27  0.27  0.27 |  |
| 6 | E-SUL-RF-CEP-TET-DOX  E-C-SUL-RF-TET-DOX  E-C-RF-CEP-TET-DOX  E-SUL-RF-TET-S- DOX  E-SUL-RF-AP-TET-DOX  E-AMC-SUL-RF-TET-DOX  E-SUL-RF-AP-CEP-DOX  E-TS-SUL-RF-AP-CEP  E-CEF-C-SUL-RF-S  E-IMI-SUL-TET-S-DOX | 3  2  1  1  2  7  2  1  1  1 | | 0.33  0.33  0.33  0.33  0.33  0.33  0.33  0.33  0.33  0.33 |  |
| 7 | E-AMC-SUL-RF-CEP-TET-DOX  E-AMC-SUL-RF-AP-CEP-DOX  E-AMK-AMC-SUL-RF-CEP-DOX  E-C-SUL-RF-CEP-TET-DOX  E-IMI-C-SUL-RF-TET-DOX  E-C-SUL-RF-TET-S-DOX  E-AMC-IMI-SUL-RF-AP-CEP  E-AMC-TS-SUL-RF-TET-DOX  E-TS-C-SUL-RF-TET-DOX  E-CEF-C-RF-CEP-TET-DOX  E-AMC-SUL-RF-TET-S-DOX  E-AMC-TS-SUL-RF-TET-DOX  E-AMC-SUL-RT-AP-TET-DOX  E-GM-C-SUL-RT-TET-DOX  E-C-SUL-RF-AP-CEP-DOX  E-AMC-IMI-SUL-RF-AP-DOX  E-AMC-IMI-C-SUL-TET-DOX  E-AMC-TS-SUL-TET-S-DOX | 4  2  1  2  1  2  1  1  1  1  1  1  1  1  2  1  1  1 | | 0.38  0.38  0.38  0.38  0.38  0.38  0.38  0.38  0.38  0.38  0.38  0.38  0.38  0.38  0.38  0.38  0.38  0.38 |  |
| 8 | E-C-SUL-RF-AP-TET-S-DOX  E-AMC-C-SUL-RF-CEP-TET-DOX  E-IMI-C-SUL-RF-CEP-TET-DOX  E-AMK-AMC-C-SUL-RF-TET-DOX  E-AMC-TS-C-SUL-RF-TET-DOX  E-AMC-C-SUL-RF-AP-TET-DOX  E-SUL-RF-AP-CEP-TET-S-DOX | 2  3  1  1  2  1  1 | | 0.44  0.44  0.44  0.44  0.44  0.44  0.44 |  |
| 9 | E-AMC-C-SUL-RF-AP-CEP-TET-DOX  E-CIP-TS-C-SUL-RF-AP-CEP-TET  E-AMC-TS-C-SUL-RF-TET-S-DOX | 1  1  1 | | 0.50  0.50  0.50 |  |
| 10 | E-AMC-IMI-C-SUL-RF-AP-CEP-TET-DOX  E-CIP-TS-C-SUL-RF-CEP-TET-S-DOX | 2  1 | | 0.55  0.55 |  |
| 11 | E-AMC-TS-C-SUL-RF-AP-CEP-TET-S-DOX | 1 | | 0.61 |  |

Keys: CIP Ciprofloxacin, AMK Amikacin, MEM Meropenem, E Erythromycin, AMC Amoxicillin, IMP Imipenem, TS Trimethoprim-Sulphamethoxazole, CEF Cefotaxime, C Chloramphenicol, G Gentamicin, SUL Sulphamethoxazole, RF Rifampin, CEP Cephalothin, AP Ampicillin, NOR Norfloxacin, TET Tetracycline, DOX Doxycycline, S Streptomycin.


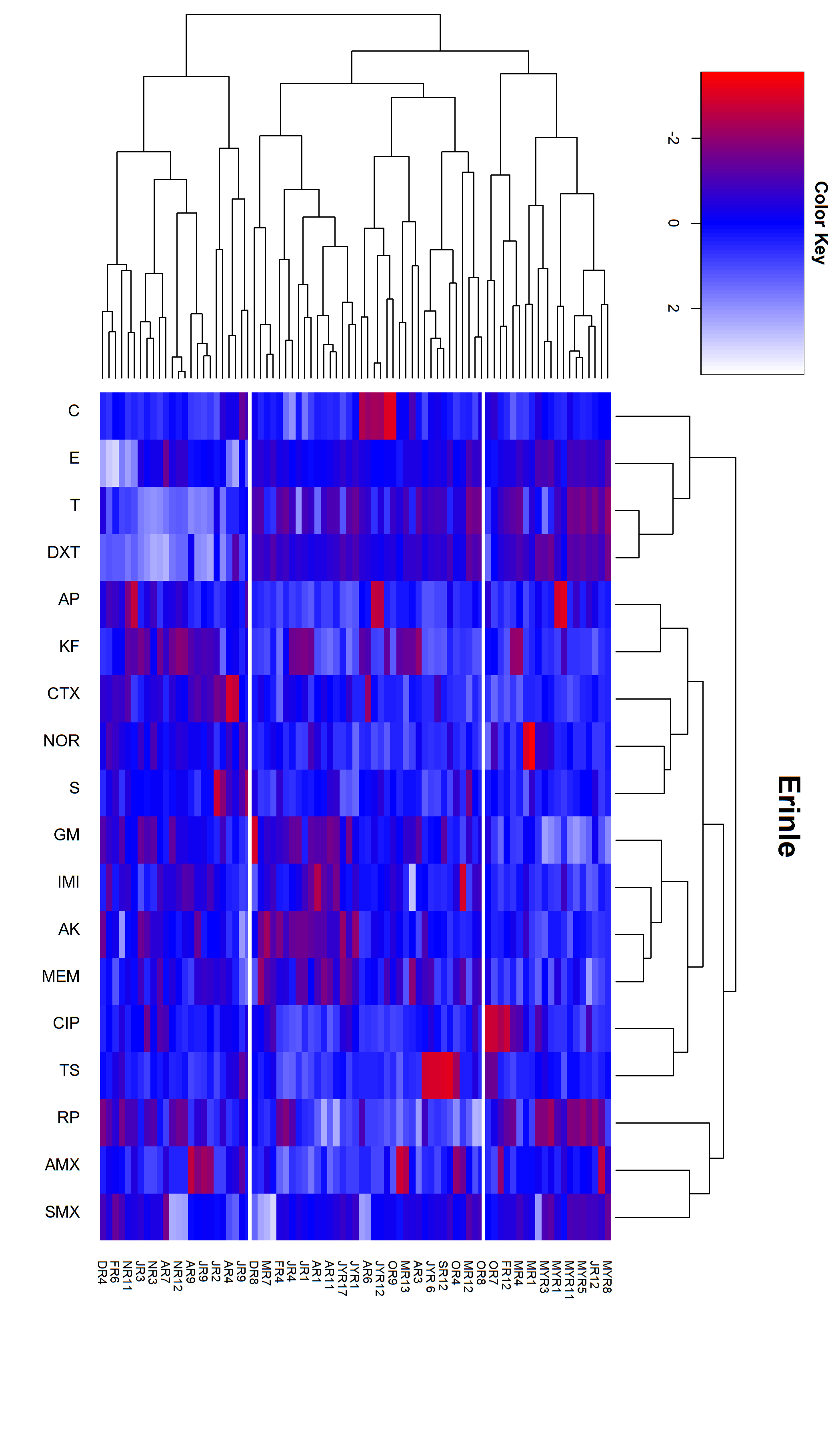


**A**


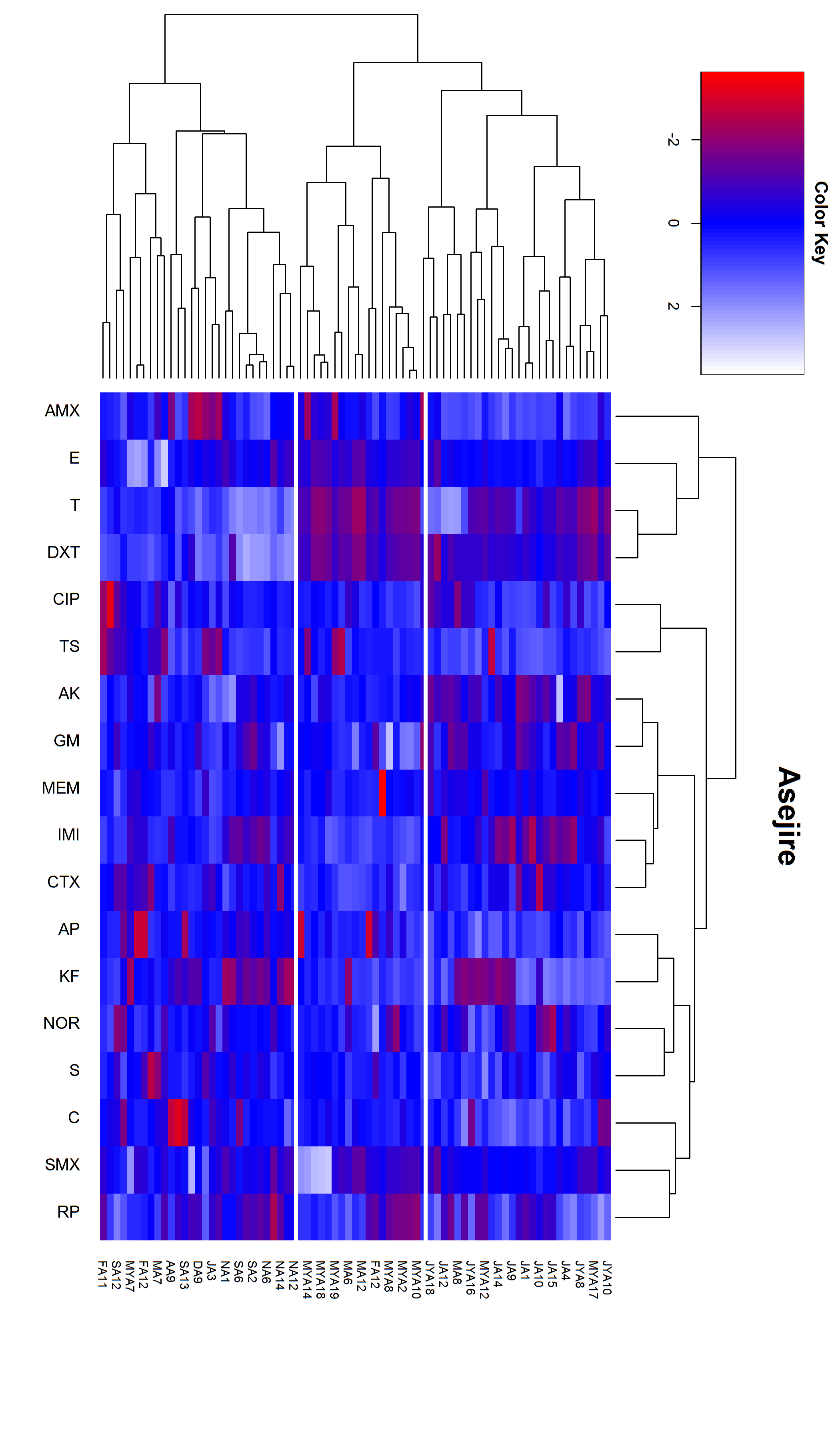


**B**


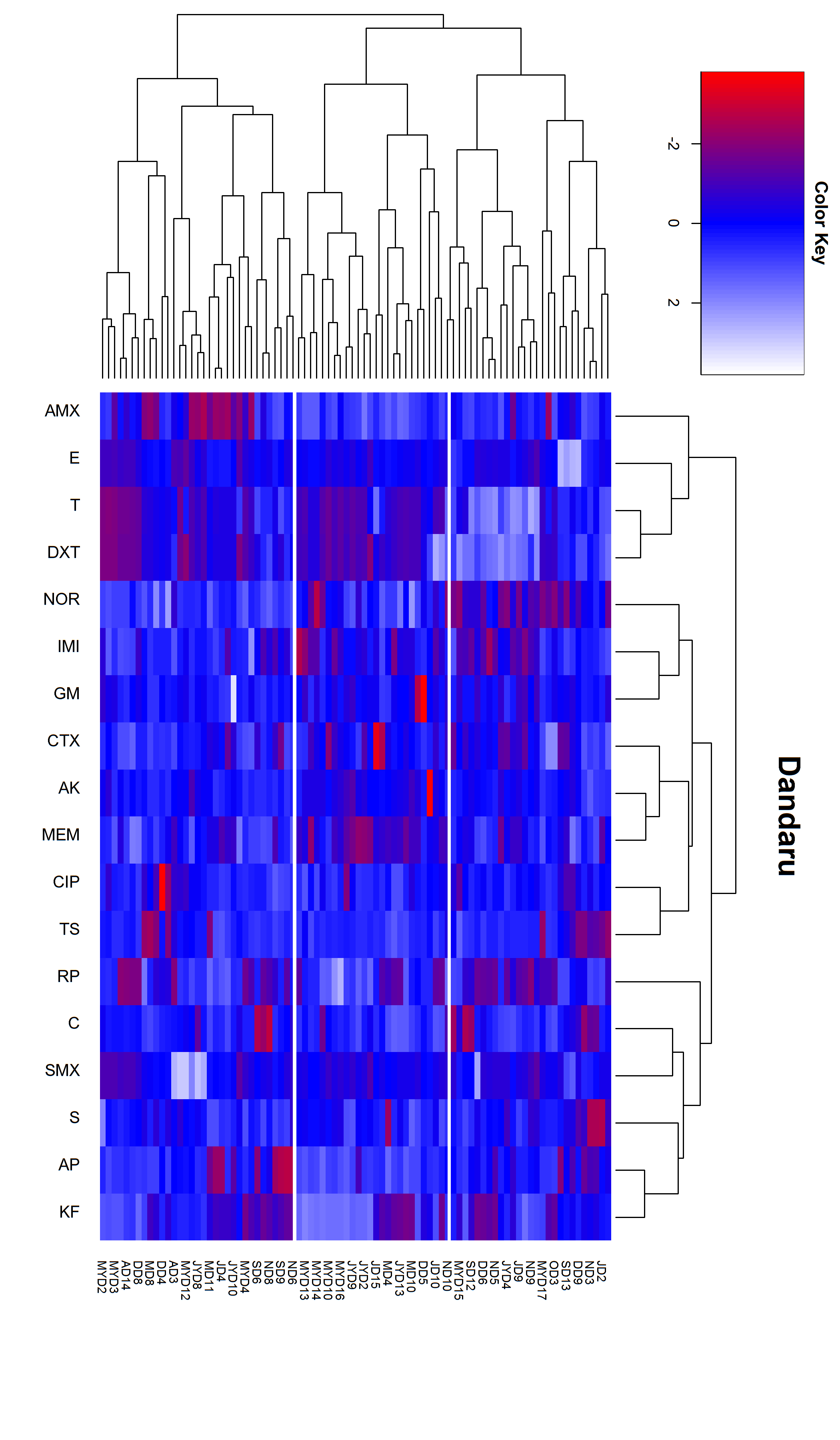


**C**


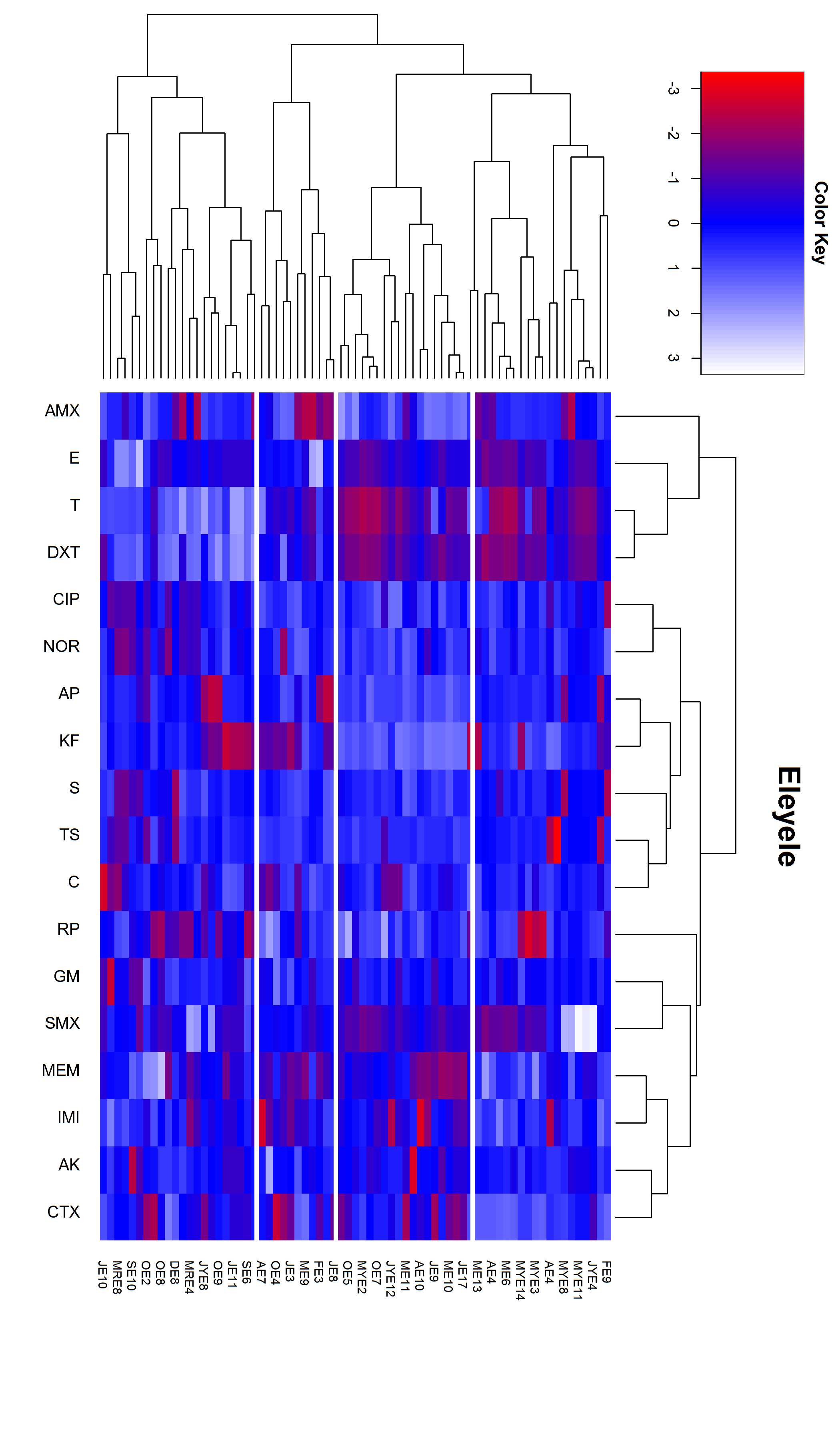


**D**

**Figure 1: Heatmap cluster of antibiogram profiles.** (A): *Vibrio* isolates from Erinle river, (B) *Vibrio* isolates from Asejire river (C) *Vibrio* isolates from Dandaru river (D) *Vibrio* isolates from Eleyele river. Colour interpretation, blue, red, white represents susceptible, resistance, and intermediate. The row and column show the clusters group for isolates and antibiotics. Antibiotic Keys: CIP Ciprofloxacin, AMK Amikacin, MEM Meropenem, E Erythromycin, AMC Amoxicillin, IMP Imipenem, TS Trimethoprim-Sulphamethoxazole, CEF Cefotaxime, C Chloramphenicol, G Gentamicin, SUL Sulphamethoxazole, RF Rifampin, CEP Cephalothin, AP Ampicillin, NOR Norfloxacin, TET Tetracycline, DOX Doxycycline, S Streptomycin,


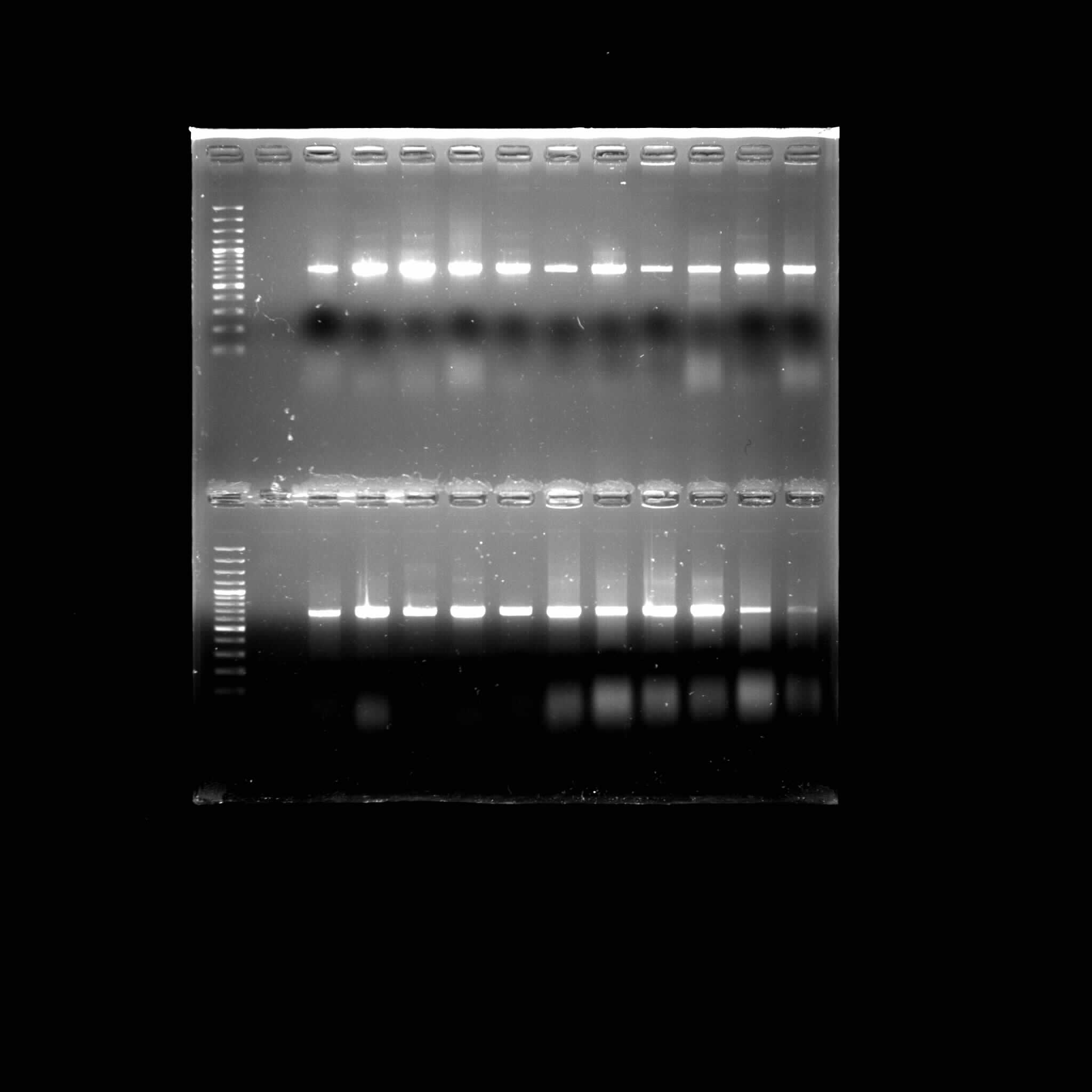


**Figure 2: PCR Product of the Amplification of *toxR* Gene for *Vibrio* Genus Confirmation.**


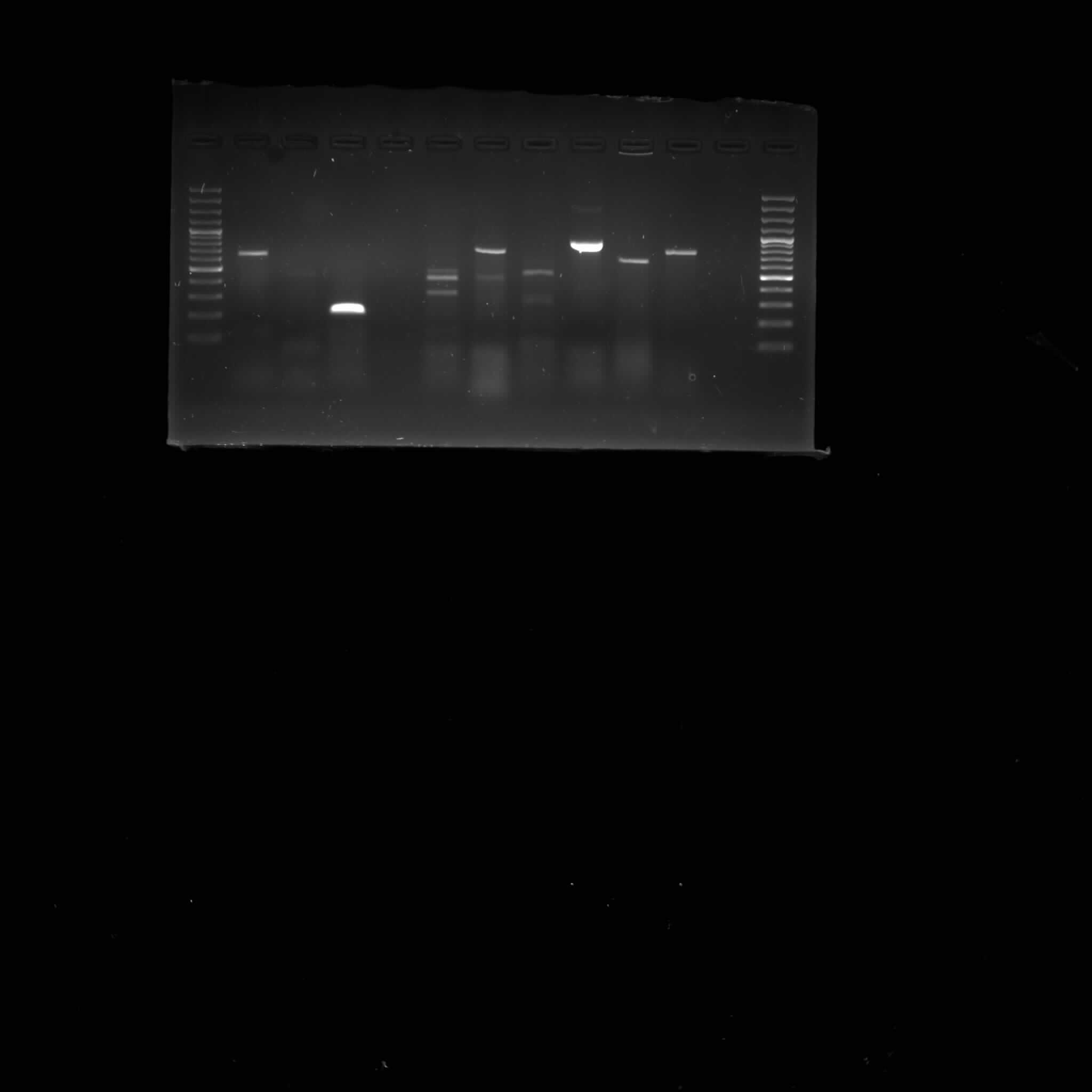


**Figure 3: PCR Product of the Amplification of the different antibiotic resistance Genes**


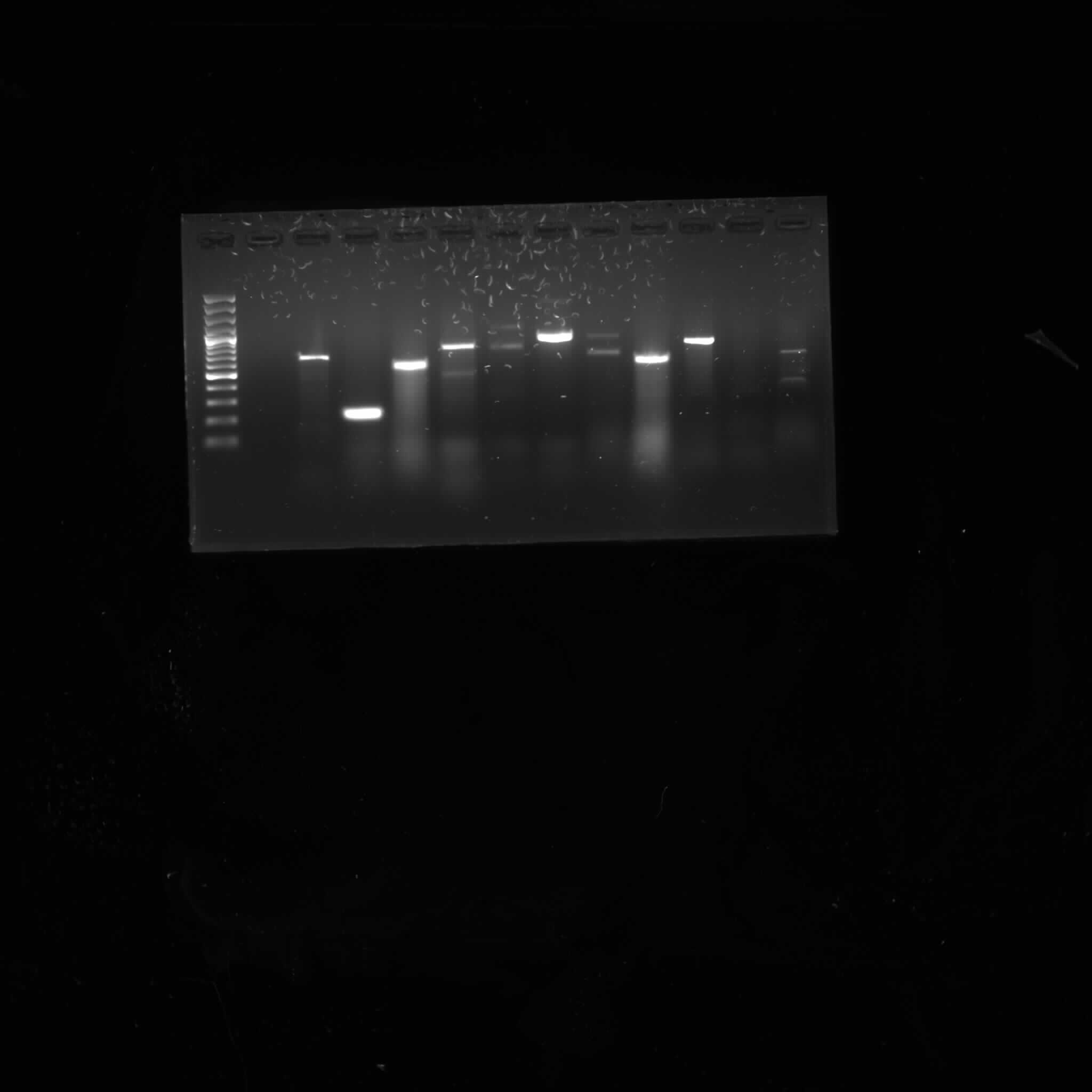


**Figure 4: PCR Product of the Amplification of the different antibiotic resistance Genes**


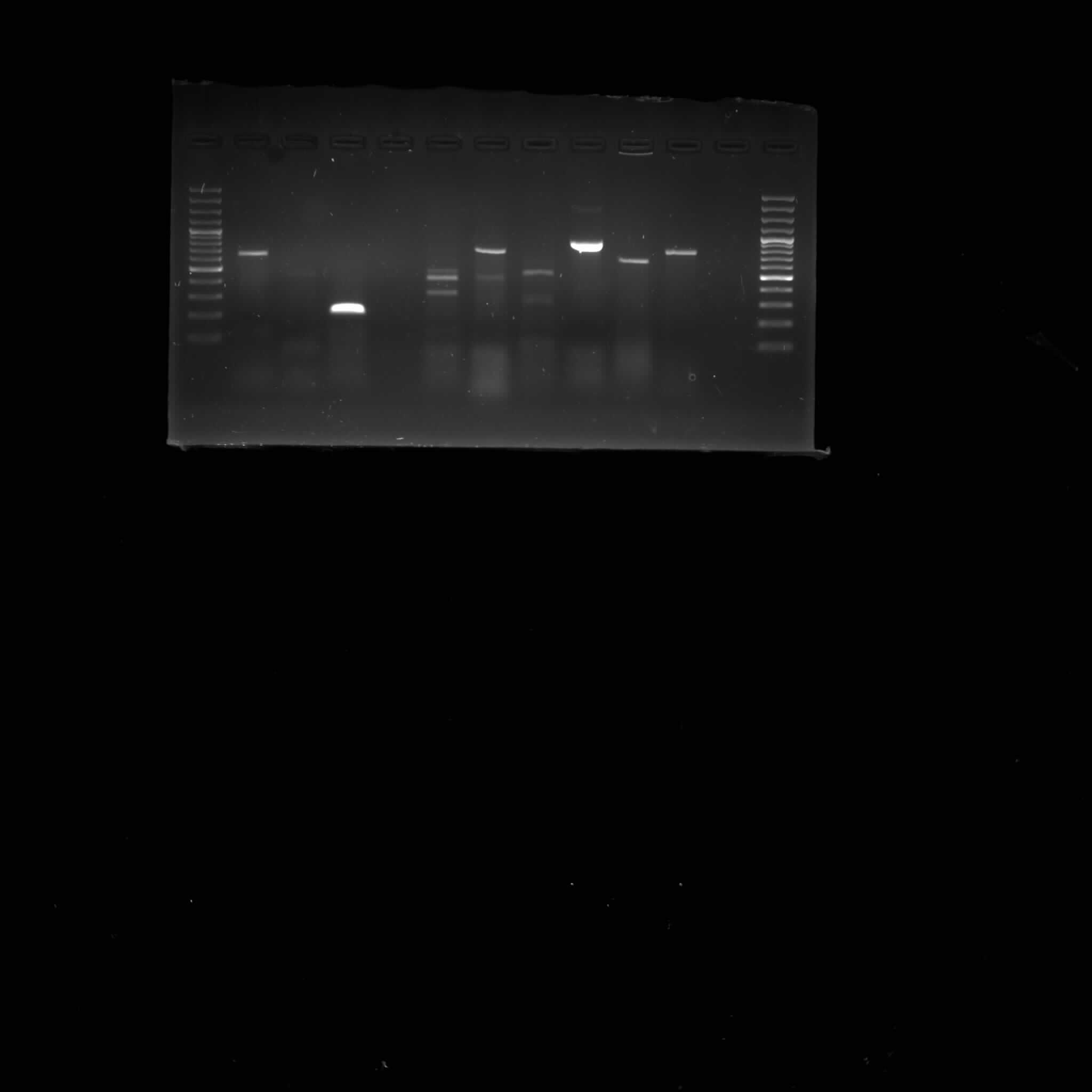


**Figure 5: PCR Product of the Amplification of the different antibiotic resistance Genes**


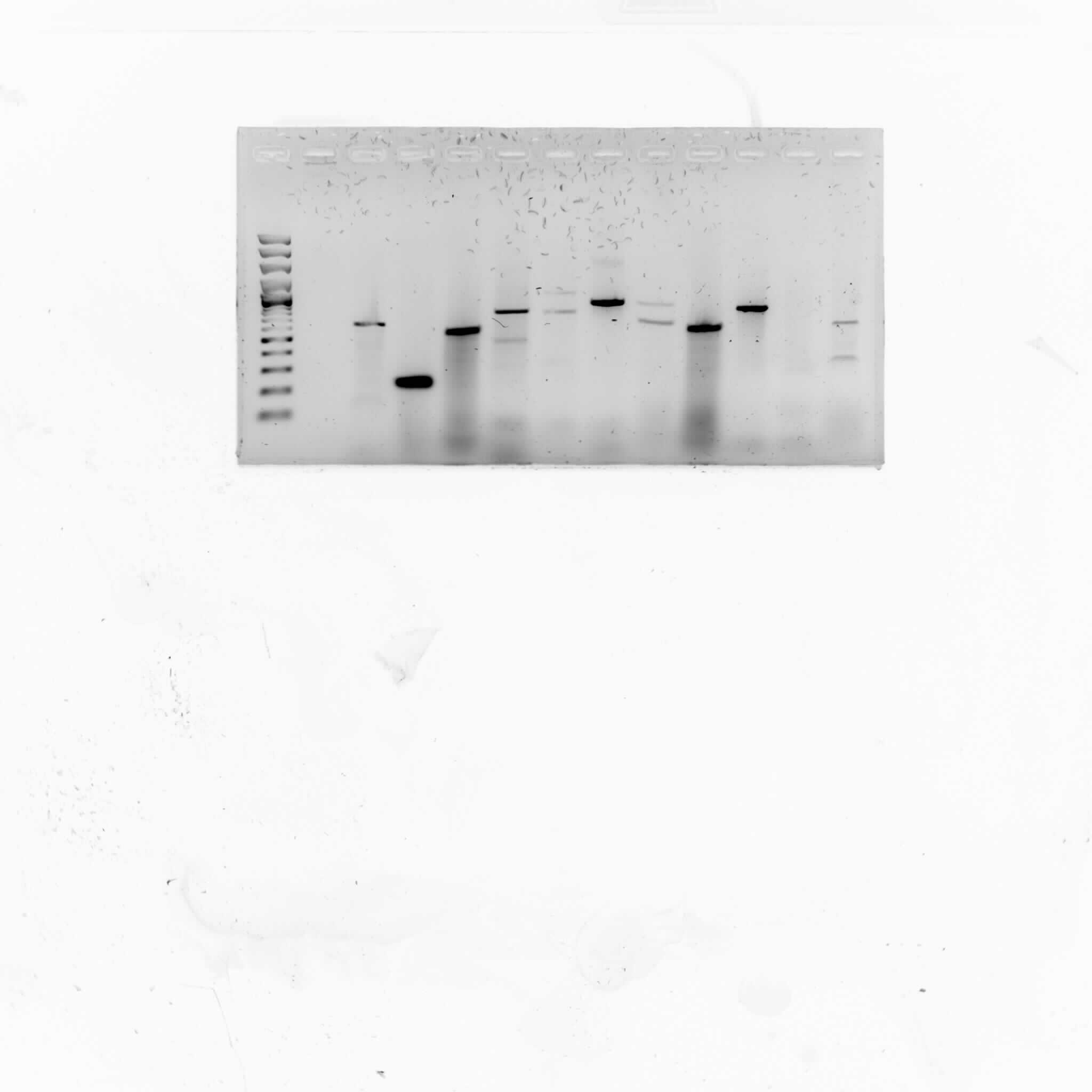


**Figure 6: Inverse of the PCR Product of the Amplification of the different antibiotic resistance Genes**
